# Unified Failure Criterion Based on Stress and Stress Gradient Conditions

**DOI:** 10.3390/ma17030569

**Published:** 2024-01-25

**Authors:** Young W. Kwon, Emma K. Markoff, Stanley DeFisher

**Affiliations:** Department of Mechanical & Aerospace Engineering, Naval Postgraduate School, Monterey, CA 93943, USA

**Keywords:** failure criterion, notch, crack, brittle, ductile, composite

## Abstract

Specimens made of various materials with different geometric features were investigated to predict the failure loads using the recently proposed criterion comprised of both stress and stress gradient conditions. The notch types were cracks and holes, and the materials were brittle, ductile, isotropic, orthotropic, or fibrous composites. The predicted failure stresses or loads were compared to experimental results, and both experimental and theoretically predicted results agreed well for all the different cases. This suggests that the stress and stress-gradient-based failure criterion is both versatile and accurate in predicting the failure of various materials and geometric features.

## 1. Introduction

Structural members are designed to avoid unexpected failure during their service life. To achieve this, these members are tested experimentally or analyzed using proper modeling and simulation techniques. Extensive physical testing is time-consuming and costly, so computational modeling and simulation are frequently used to replace or minimize unnecessary testing. In order to have confidence in the accuracy of the results, however, computational modeling should be reliable. To this end, many failure theories have been developed to predict failure loads based on material type, including isotropic and anisotropic ones, subjected to a variety of loading conditions, including static and cyclic scenarios [1].

Important data in designing load-carrying structural members include the maximum load that they can carry without failure. Hence, failure criteria are necessary to predict the maximum failure load, but the load also depends on the geometry and material of the structure. If the structures have notches such as holes or cracks, their load-carrying capacity is significantly limited.

In the past, different failure theories were used to predict failure loads of structural members depending on the state of notches they contained. For example, many different failure criteria were proposed for structural members without any notches and subjected to combined loading (i.e., multiaxial loading) [1,2,3,4,5,6,7,8]. Those criteria were to apply the failure strength obtained from uniaxial testing to the prediction of failure under combined loading. However, if a structural member has a notch, those failure criteria are not reliable.

A structural member with a crack has stress singularity at the crack tip if the material behaves linearly elastically. Thus, fracture mechanics was also developed for structural members with cracks [9,10,11,12,13,14,15,16]. On the other hand, if structural members have holes, an entirely different set of failure criteria was used because fracture mechanics is suitable for holes. The critical distance failure criteria, for example, is often applied for structural members containing holes [17,18,19,20,21,22,23,24,25]. Some used the stress at the critical distance, while others used the average value up to the critical distance from the notch tip to predict failure at the notch tip.

The cohesive zone model was also developed to predict failure loads better [26,27,28,29,30,31,32,33]. The model considers a localized zone around the potential failure location, which is called the cohesive zone. A traction–separation relationship is applied to the cohesive zone and is used to predict failure. For the cohesive zone model to be accepted as a failure theory, the same traction–separation relationship must be applicable to structural members independent of the notch shape, like a crack or a hole, as well as its size. 

Recently, a unified failure criterion was proposed by the authors’ team [34,35,36,37]. The unified failure criterion can be applied to structural members regardless of the existence of notches, as well as their shapes and sizes. This failure criterion was validated against different experimental data on brittle specimens with cutouts, including holes and slits. The objective of this study is to validate the unified failure criterion further to determine whether the theory can apply to various cases, which include ductile or brittle materials; isotropic materials, 3D-printed orthotropic materials, or laminated fibrous composites; and holes, long slits, or cracks. The next section describes the new unified failure criterion, and it is followed by various sections that discuss the failures of different cases with subsequent conclusions.

## 2. Failure Criterion Based on Stress and Stress Gradient

The recently proposed failure criterion uses both stress and stress gradient to determine failure. Both stress and stress gradient conditions must be satisfied for failure to occur [34,35,36,37]. First, the stress condition must be checked. This condition states that the effective stress at any material point should not be less than the failure strength of the material, which is stated below:

(1) $$\sigma_{e}\geq \sigma_{f}$$

where $\sigma_{e}$ and $\sigma_{f}$ are the effective stress and failure strength, respectively. The effective stress is different depending on the material behavior. The maximum normal stress is usually used for the effective stress for an isotropic brittle or quasi-brittle material. On the other hand, the maximum shear or the octahedral shear stress is selected as the effective stress for an isotropic ductile material. If a material is anisotropic, multiple effective stresses are considered depending on the directions of the material properties and the loading.

Once the stress condition is satisfied, the stress gradient condition must also be checked. This condition is expressed as

(2) $$\sigma_{e}\geq {\left(2E\Upsilon \left(\frac{d\sigma_{e}}{ds}\right)\right)}^{1/3}$$

where *E* is the modulus; Υ is a material failure value, which is discussed below; and *s* is the failure path. For a brittle material, the failure path is normal to the maximum principal axis at the failure location. When both stress and stress gradient conditions are satisfied at any point in the sample in question, failure is deemed to have occurred. In other words, even if the effective stress is greater than the failure strength, failure does not occur unless the stress gradient condition is also satisfied.

To investigate the material failure value Υ, which is different from $\sigma_{f}$, we consider a crack under the first mode of fracture. The stress field very near a crack is expressed as

(3) $$\sigma_{e}=\frac{K}{\sqrt{s}}$$

in which *s* is along the crack orientation measured from the crack tip, which is perpendicular to the loading direction. Equation (3) and its derivative are substituted into Equation (2), which, at the onset of failure, results in the following:

(4) $$\Upsilon =\frac{K^{2}}{E}$$

Thus, the material failure value Υ is equivalent to the critical energy release rate in fracture mechanics.

## 3. Ductile Aluminum Alloy with Notches

### 3.1. Description of Specimens

**Figure 1:**
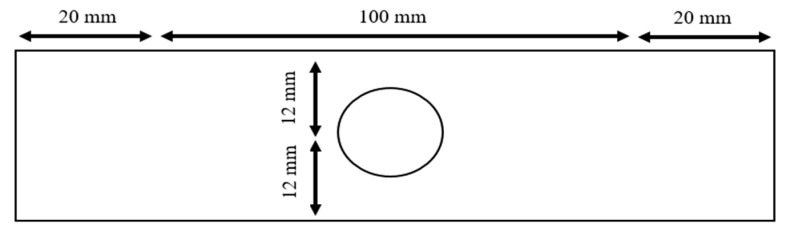
Aluminum specimen with a center hole.

**Figure 2:**
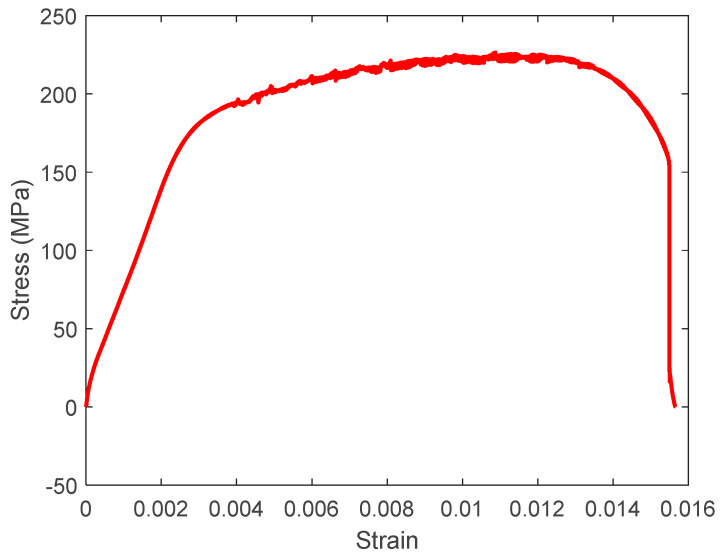
Stress–strain curve of 5000 series aluminum alloy.

All test specimens were 140 mm long, 24 mm wide, and 1 mm thick. The grip-to-grip distance was 100 mm, meaning that each specimen was captured over 20 mm on each end. Any notch was introduced at the center of every specimen, as sketched in Figure 1. The size of the circular hole varied from 1 to 18 mm in diameter incrementally. All the holes were located at the center of the specimens. There were six specimens for every size of the hole. All the specimens were tested under tensile loading using INSTRON 5982 (Norwood, MA, USA). In addition, six dog-bone shapes of specimens were also tested, without any notch, to determine the stress–strain curves of the 5000 series aluminum alloy. Strain gauges were attached to the dog-bone shape of specimens to measure both longitudinal and transverse strains. Figure 2 shows the stress–strain curve of the aluminum alloy. The graph shows the very ductile nature of the material with a low tangential modulus for strain hardening.

**Figure 3:**
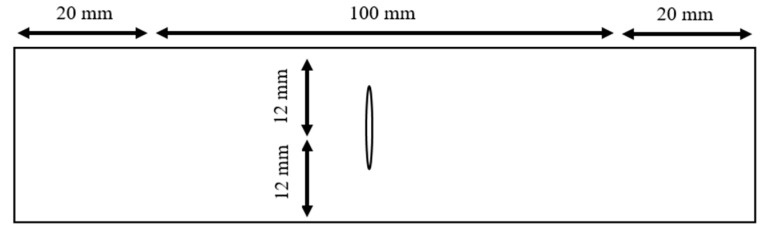
Aluminum specimen with a center slit.

The next set of specimens had the same dimensions as before, but a slit was introduced at the center instead of a circular hole, as sketched in Figure 3. The slit length was either 4 mm or 6 mm. The 6 mm slit had four different orientations with respect to the width direction of the specimens. The orientation angles were 0°, 15°, 30° and 45°, respectively. Each type of specimen with a slit was called Lx/y°, where x is the crack length in mm and y is the orientation angle in degrees. Six of every specimen type were prepared for testing.

### 3.2. Results

**Figure 4:**
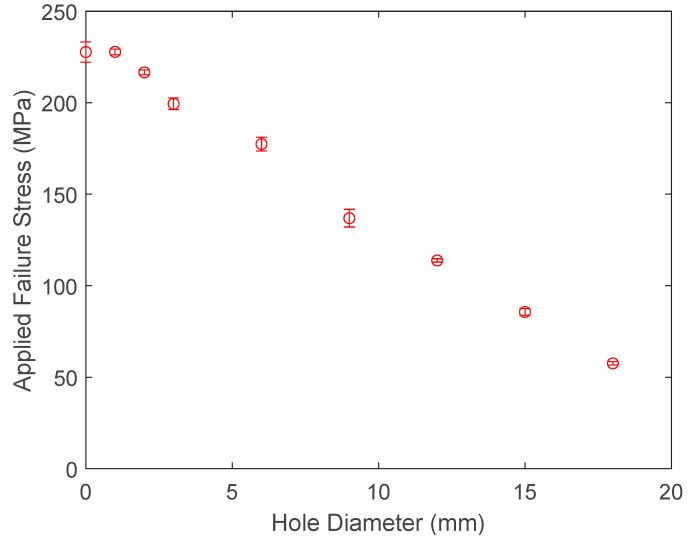
Applied failure stress of aluminum specimens with different hole sizes.

Tensile tests of the dog-bone-shaped specimens, as well as the specimens with variable diameters of center holes, were conducted to determine their failure loads. Then, the applied failure stresses were computed from the failure loads divided by the cross-sections at the grips, which do not consider the hole. The applied failure stresses are plotted in Figure 4 for different hole sizes. The plot shows that the applied failure stresses decrease almost linearly as a function of the hole diameter, and the standard deviations of the notched specimens were very small as compared to that of the dog-bone-shaped specimens. In addition, the specimens with a hole of a 1 mm diameter had applied failure stresses almost the same as that of the dog-bone specimens, even though the dog-bone-shaped specimens had a larger standard deviation than the perforated specimens. The dog-bone-shaped specimen data were included in the figure if the failure occurred at the mid-section of the specimens. Otherwise, the data were excluded. Figure 4 confirms that a very small hole, as compared to the specimen width, does not have a noticeable effect on the load-carrying capacity, and the load-carrying ability decreases as the hole size further increases.

The data in Figure 4 were re-calculated such that the applied failure stress was replaced by the nominal failure stress at the minimum cross-section across each hole. The nominal failure stress is the average stress across the minimum cross-section at the onset of failure. Table 1 shows the results. It is reasonable to state that the nominal failure stress was almost constant, independent of the hole size. This suggests that for this geometry, the stress failure condition dominates the failure of the aluminum test specimen. Therefore, the stress gradient condition was easily satisfied because the failure value from the stress gradient condition is much smaller than that from the stress condition. In order to demonstrate this, the stress gradient was computed at the edge of the hole of different sizes using finite element analysis to investigate the stress gradient condition. 

Specimens of 100 mm × 24 mm with a center hole were modeled for the plane stress condition using four-node quadrilateral elements in Ansys [38]. The elastic–plastic analysis was conducted using the stress–strain curve obtained from the dog-bone-shaped specimen. Because the tangential modulus during the plastic deformation is quite small compared to the elastic modulus, the stress gradient at the edge of the hole becomes smaller, along with more plastic deformation around the edge of the hole. As a result, the stress gradient with the ductile aluminum alloy becomes much smaller than that of any elastic analysis of brittle materials. That is, the stress gradient of the former was at least an order of magnitude less than that of the latter. Thus, the stress gradient condition was already satisfied at the edge of the holes. This indicates that the failure of the ductile aluminum specimens with a low tangential modulus of plastic deformation was also governed by the stress condition rather than the stress-gradient condition, even though they contain a circular hole. 

**Figure 5:**
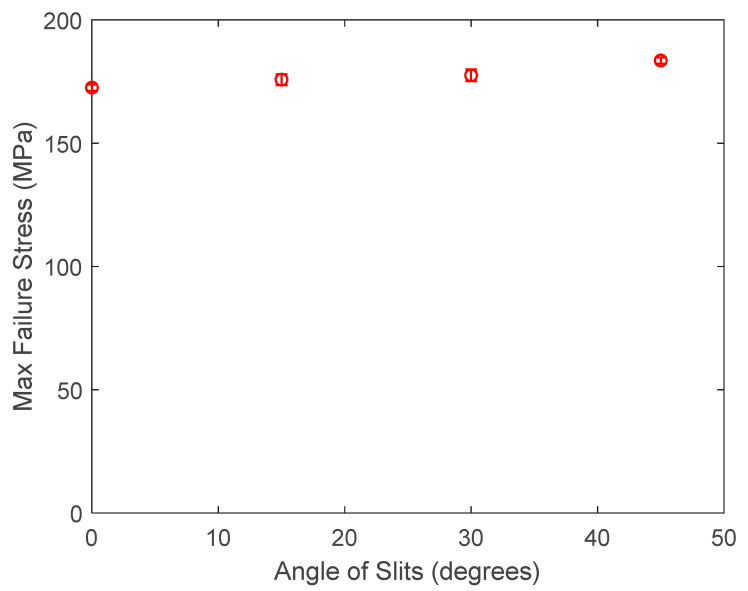
Plot of applied failure stress vs. the slit angle of slit size of 6 mm.

**Figure 6:**
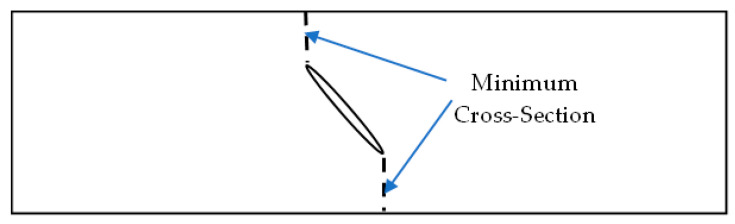
Minimum cross-section for a slit with an orientational angle.

**Figure 7:**
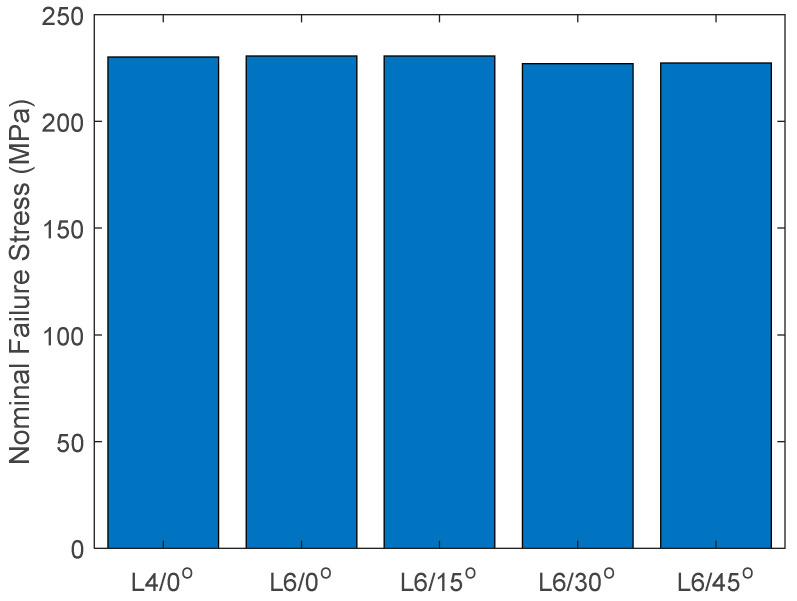
Nominal failure stress of aluminum specimens with different slit lengths and angles.

The aluminum specimens containing slits were also plotted in Figure 5 for the applied failure stress. This stress was calculated by dividing the failure load divided by the cross-sectional area without considering the slit. The results show an increase in the failure stress along with the slit angle. Then, the nominal failure stresses were computed across the minimum cross-section of the specimen. This minimum cross-section is defined in Figure 6 for slits with nonzero orientation angles. The nominal failure stresses were also almost constant for different slit lengths and orientations, as seen in Figure 7. This also indicates that the failure of the specimens with slits was predicted exclusively by the stress failure condition instead of the stress gradient failure condition, as explained for the aluminum specimens with circular holes. In other words, the stress gradients were so small that the failure stress resulting from the stress gradient condition was smaller than the failure stress from the stress condition. Thus, the failure stress from the stress condition is the failure strength of the specimen.

## 4. Hardened Cement Pastes with Cracks

### 4.1. Description of Specimens

**Figure 8:**
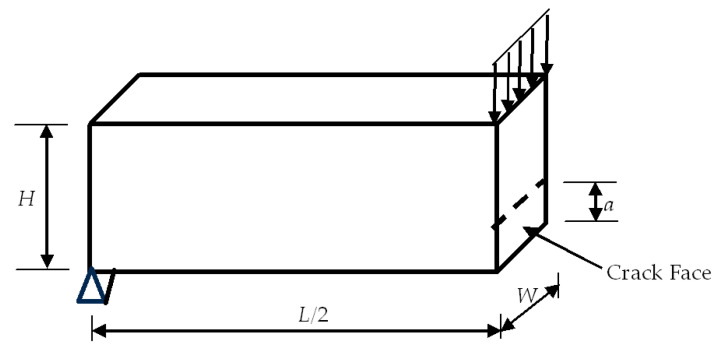
Half of the hardened cement paste model.

The next set of specimens was hardened cement paste with cracks, which were studied experimentally in Ref. [39]. All the specimens are 3-dimensional blocks of *L* mm × *H* mm × *W* mm, where *L* is the length, *H* is the height, and *W* is the width of the specimen. Each specimen has a crack of the depth ‘*a*’ in the middle of the length and at the bottom side. The crack is through the width of every specimen, which was tested under the three-point bending setup. Figure 8 shows half of the hardened cement paste model because of symmetry. 

All the specimens made of the hardened cement paste had the width *W* = 100 mm, and the length to the depth ratio remained as *L*/*H* = 4 while the depth was varied with different ratios of the crack to specimen depth *a*/H. The hardened cement paste behaved in a brittle manner with an elastic modulus of 20.8 GPa.

### 4.2. Modeling

**Figure 9:**
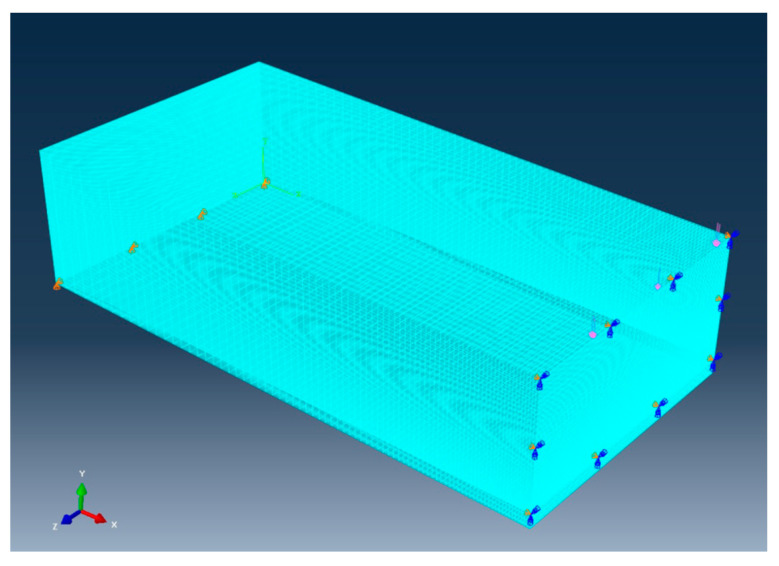
Three-dimensional three-point bending model of hardened cement paste.

First, half of the specimen, as sketched in Figure 8, was modeled using 3D solid elements. The mesh was uniform, with the element length around 0.3 mm. The supporting and symmetric boundary conditions were applied to the model, as well as the applied load across the width of the specimen. Figure 9 shows the 3D finite element mesh of the model with boundary and loading conditions. The right face had a symmetric boundary condition except for the crack face at the bottom side.

After linear elastic analysis, the stress profiles were examined along the crack line across the width of the specimen. The results showed that the stress variation from the edge of the crack to the vertical direction was very close across the specimen width. This suggests that 2D analysis would be acceptable to save computational time. Therefore, 2D analyses of three-point bending were conducted using four-node quadrilateral elements to predict the failure loads of the specimens made of the hardened cement paste. After some mesh sensitivity study, the final mesh was around 10,000 elements.

### 4.3. Results

The failure loads were predicted for the hardened cement paste specimens with initial cracks from the 2D FEA, as discussed in the previous section. Because of the crack with stress singularity, the stress gradient condition is used for the prediction of the failure loads. In other words, the stress condition is already satisfied at the crack tip. 

**Figure 10:**
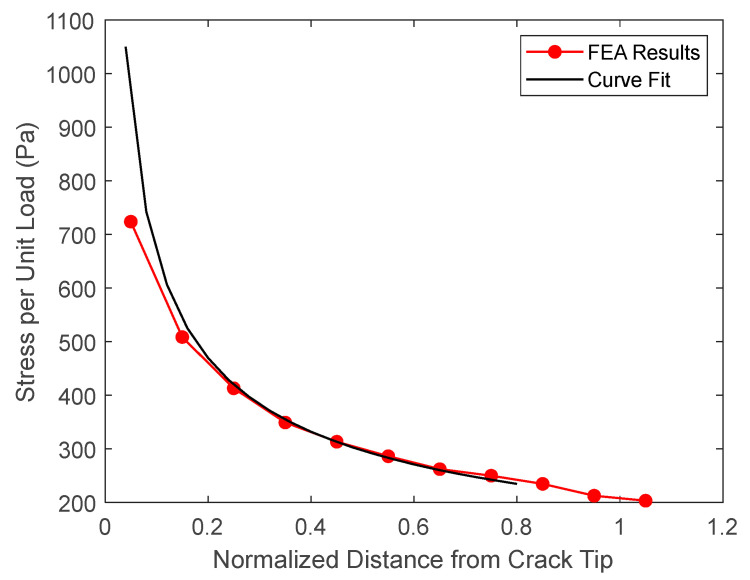
Curve fit of the FEA solution to determine the stress intensity factor.

Since the failure value Υ, which is related to the critical energy release rate, is not known for the given material, one of the test results in Ref. [39] was used to extract the value. Then, the same failure value Υ was used for the remainder of the specimens to predict the failure loads. The stress intensity factor *K* was obtained from 2D finite element analyses. Because conventional FEA, in general, does not provide accurate stresses very near the crack tip, a curve fit was conducted using Equation (3) to determine the stress intensity factor *K* for a given applied load. Figure 10 shows an example of the curve fit to the FEA solution. Then, the failure loads are determined using Equation (4).

**Figure 11:**
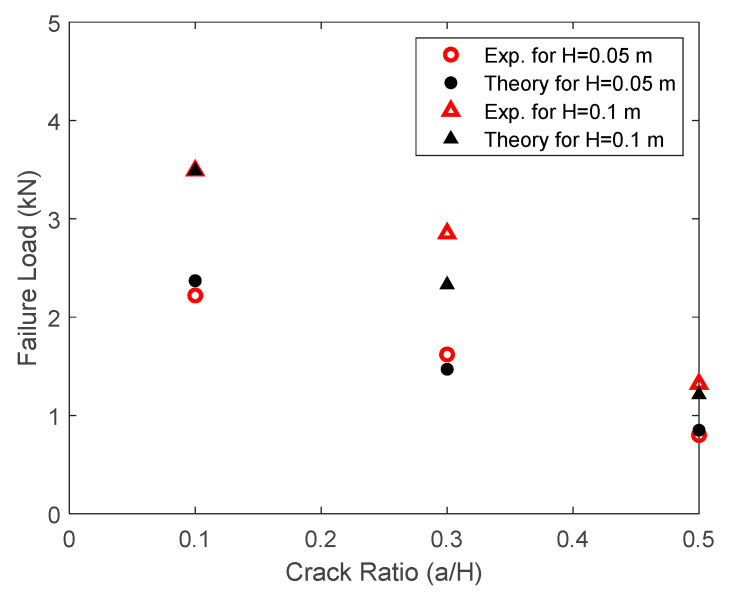
Comparison of failure loads of hardened cement paste specimens with cracks.

Figure 11 shows the comparison between the predicted failure loads and the experimentally measured values. Because the specimen with a crack-to-depth ratio of 0.1 and the specimen height of 100 mm was used to determine the failure value Υ, both theoretical and experimental values agreed exactly for the specimen in this case. All other specimens show close agreement between the two results except for one specimen with a crack ratio of 0.3 with a specimen height of 100 mm. Because the authors do not have additional information on that test specimen, no further study could be conducted to understand the difference between the two results. Overall, the results confirmed the applicability of the present failure criterion to predict the failure of cracked specimens. 

## 5. Three-Dimensional Printed PLA with Holes

### 5.1. Description of Specimens and Experiments

PLA specimens were printed by a 3D printer using the fused filament fabrication technique. The printing conditions influence the material properties of the 3D-printed PLA specimens. For example, the printing temperatures affected the strength of the printed specimens. In other words, the strength in the printing direction was much greater than that in its transverse direction, such that the 3D printed specimens behaved like an orthotropic material, such as a unidirectional fibrous composite. 

The printing conditions for the present PLA specimens are provided in Table 2. First, rectangular shapes of specimens were printed in 0°, 90°, and ±45° with a length of 140 mm, a width of 24 mm, and a thickness of 2 mm. The printing angle is with respect to the length direction of the specimens, which is also the loading direction. This means that the 0° specimens were printed along the loading direction, and those specimens were stronger than other specimens. For uniaxial testing, tabs of 20 mm × 24 mm were attached to both sides of every specimen on both ends. That is, each specimen had four tabs so as not to fail at the grip section during the tests. This made the gauge length of every specimen 100 mm.

**Figure 12:**
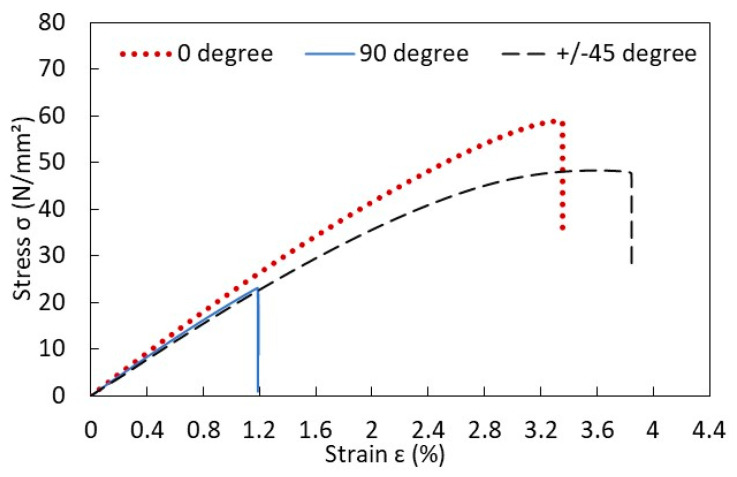
Stressstrain curves of rectangular PLA specimens.

The stiffness and strength were determined from the tensile tests of the rectangular shapes of specimens with tabs. Figure 12 shows the typical stress–strain curves of the PLA specimens printed in different orientations relative to the loading direction. The test results indicate a larger difference in strength than in stiffness. Those graphs provided the material properties of the PLA specimen. To determine Poisson’s ratio, strain gauges were also attached to the specimens in both longitudinal and transverse directions. The details are given in Ref. [37].

**Figure 13:**
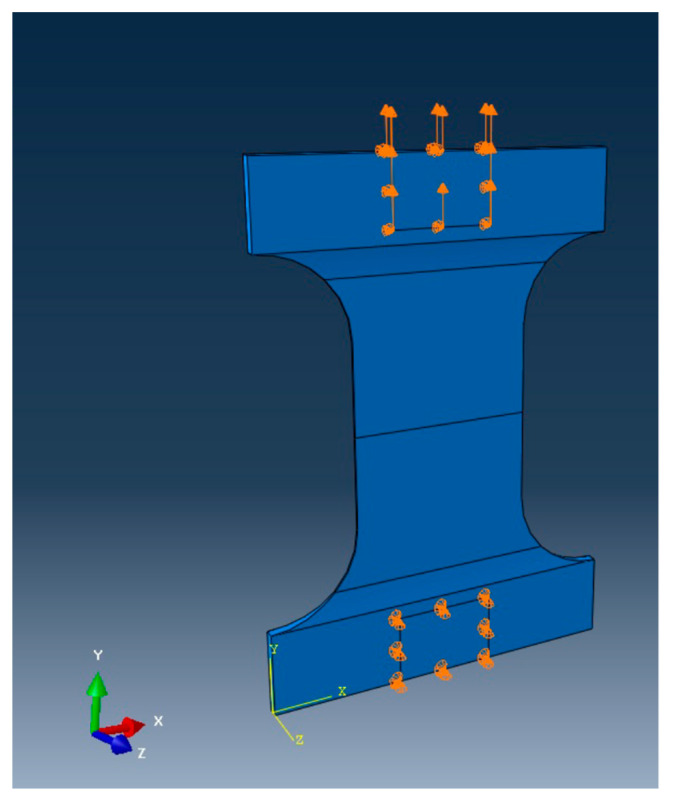
Dog-bone shape PLA specimens.

In the previous study [37], rectangular specimens with the same geometry were tested with different sizes of holes and different printing angles relative to the loading direction. Then, the experimental results were compared to the predicted failure loads. The agreement was very good. In this paper, much larger sizes of dog-bone-shaped specimens were printed using PLA. Those specimens were tested to investigate the effect of the hole size relative to the specimen width on the failure load. Figure 13 shows the dog-bone shape of a PLA specimen. 

All the dog-bone shapes of PLA specimens were printed along the loading direction, i.e., at a 0° angle. All specimens had a test section of 80 mm wide and 1 mm thick. The top and bottom portions of the specimen were 3 mm thick, while the thickness gradually decreased to 1 mm in the test section of the specimen. This is to prevent failure around the grip sections of the specimens because the testing equipment could hold only a small portion of the end sections, as sketched in Figure 13. In other words, there was no grip available for the testing, which was wide enough to hold the whole width of the specimen. The hole was drilled at the center of every specimen, and the hole size was 3 mm, 4 mm, 5 mm, or 6 mm, respectively. At the minimum, three specimens were tested for the same size of the hole. 

All the dog-bone shapes of PLA specimens were subjected to tensile loading until failure. The maximum forces were obtained as the failure loads from which the applied stresses at failure were computed. Furthermore, a high-speed video was used to capture the locations of the initial failures of the specimens. The video was set to 50,000 frames per second. The observed failure locations were later compared to the predicted failure location.

### 5.2. Results

The dog-bone specimens were modeled using 2D quadrilateral elements only for a quarter of their geometry because of double symmetries. To emulate the physical test condition, uniform displacements were applied to the FEA model at the grip section of each specimen, as shown in Figure 13. The applied displacement was increased gradually until both stress and stress-gradient failure conditions were satisfied at any material point that would be the location of the initial failure. The analyses were conducted for specimens with different hole sizes. 

**Figure 14:**
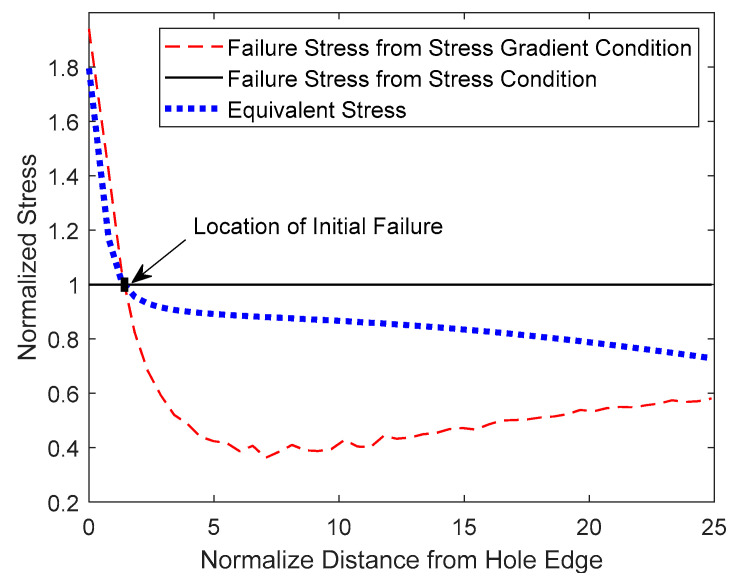
Analytical failure prediction of a PLA dog bone with a 3 mm diameter center hole.

**Figure 15:**
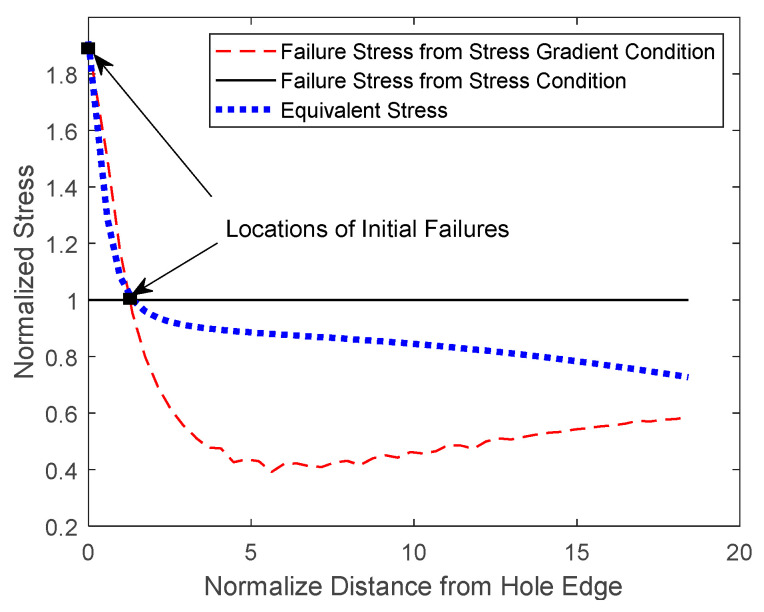
Analytical failure prediction of a PLA dog bone with a 4 mm diameter center hole.

**Figure 16:**
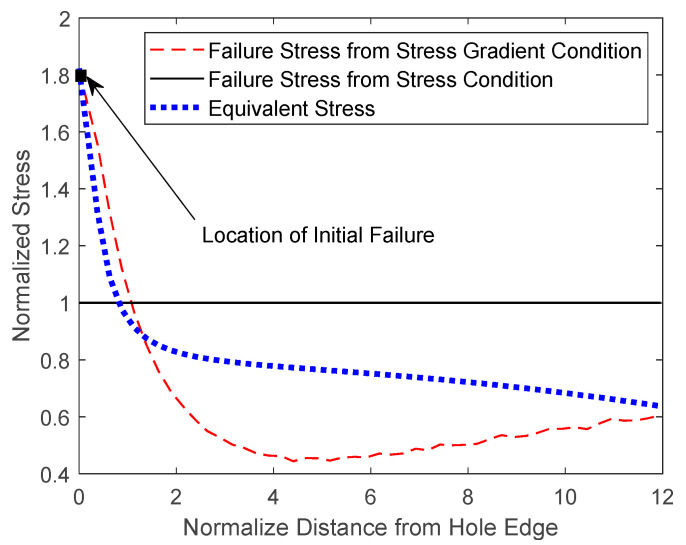
Analytical failure prediction of a PLA dog bone with a 6mm diameter center hole.

Figure 14, Figure 15 and Figure 16 show both the analytical predictions of failure locations for the 3, 4, and 6 mm holes, respectively. Each figure has three graphs. The first graph is the failure strength from the stress failure condition, the second graph is the failure strength from the stress-gradient condition, and the third graph is the induced effective stress from the applied loading. All the graphs were normalized with respect to the failure strength from the stress condition. The failure strength from the stress condition is constant, independent of the location in the specimen. However, failure strength from the stress-gradient condition, as well as the induced equivalent stress, varies across the specimen from the hole. 

The FEA analysis gave the failure strength computed from the stress-gradient condition at every node or element starting from the edge of the hole. The stress-gradient-based failure strength varies at different locations because the stress gradients change from point to point. Those varying failure stresses from the stress-gradient condition are plotted in the figures along the minimum cross-section of the specimen from the edge of each hole. 

The equivalent stress is the maximum normal stress along the same minimal sections of the specimens. The equivalent stress increases as the applied displacement to the specimen models increases. For the failure to initiate, the equivalent stress at one location must be equal to or greater than both failure stresses from the stress and stress-gradient failure conditions. Figure 14, Figure 15 and Figure 16 show the plots of the equivalent stresses that just meet both failure conditions at the initial failure locations for different sizes of the center holes from 3 mm to 6 mm.

Figure 14 is for the 3 mm hole, which shows that the equivalent stress meets both failure strengths from the stress and stress-gradient conditions, respectively, at a distance away from the edge of the hole. Before that failure location, the stress-gradient condition is not satisfied, and after the location, the stress condition is not satisfied. Thus, the initial failure does not occur at the edge of the 3 mm hole but rather some distance away from it. Hence, the stress concentration at the hole edge did not influence the failure, and the failure load was not affected by such a small hole in the specimen.

When the hole size was increased to 4 mm, Figure 15 shows there are two potential failure locations: one at the edge of the hole and the other at a distance away from it. That is, both stress and stress-gradient conditions could be satisfied at both locations almost simultaneously. This suggests that the failure would occur at one site, immediately followed by the other site. On the other hand, failure occurs at the edge of the hole when the hole size grows to 6 mm, as shown in Figure 16. 

**Figure 17:**
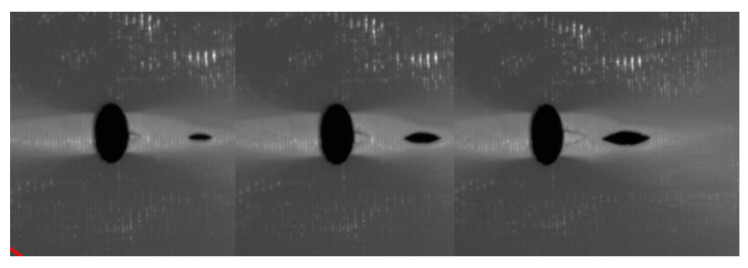
Progression of initial failure of the specimen with a 3 mm hole.

Experiments were conducted using PLA specimens with different hole sizes. As the tensile load was applied to each specimen until failure, a high-speed video was used to capture the moments of initial failures. Figure 17 shows the video clip of the failure progression of the specimen with a 3 mm hole just after the initial failure. The experiment agrees with the theory showing the failure initiation away from the edge of the 3 mm hole.

**Figure 18:**
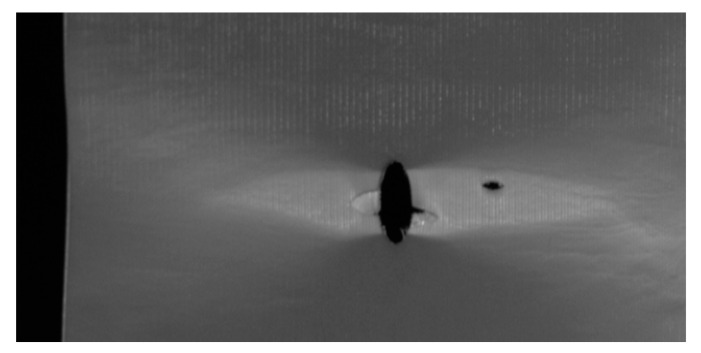
Progression of initial failure of the specimen with a 4 mm hole.

**Figure 19:**
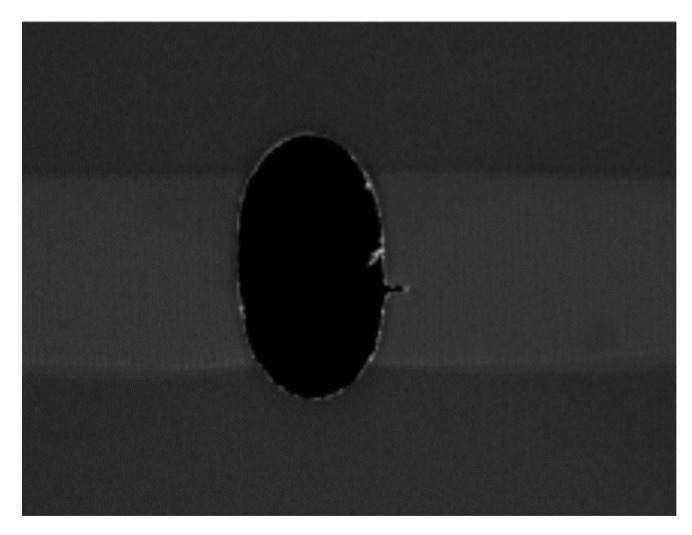
Initial failure of the specimen with a 6 mm hole.

For the 4 mm hole size, some specimens showed initial failure starting from the edge of the hole, and others showed it at a distance away from the edge. Figure 18 shows the first failure at the edge of the hole, which was followed immediately at a distance away from the 4 mm diameter hole. On the other hand, the specimen containing a 6 mm central hole showed failure initiation from the edge of the hole, as seen in Figure 19. Because of uncontrollable asymmetry, the initial failure occurred on one side of the hole, and then failure also followed on the other side of the hole. Thus, the experimental results confirmed the theoretical predictions based on both stress and stress-gradient failure criteria.

**Figure 20:**
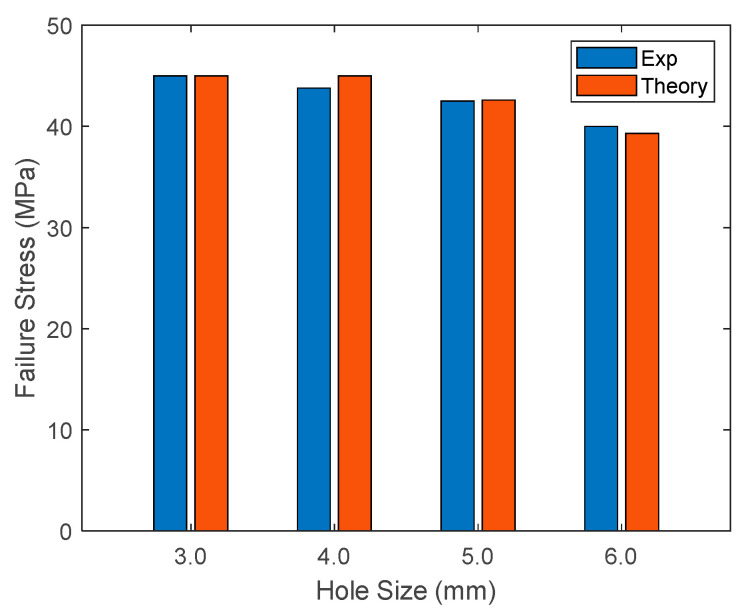
Comparison of theoretical and experimental failure stresses of PLA specimens with different hole sizes.

The applied failure stresses of the PLA specimens at the onset of failure were also compared between the theory and the experimental results. Figure 20 shows this comparison. Both results agreed well with each other.

## 6. Laminated Glass Fiber Composites with Holes

### 6.1. Description of Specimens

Test specimens were cut out of a quasi-isotopically laminated glass fiber composite (GFC) plate, which has the following layer angles: 0°/45°/−45°/90°/90°/−45°/45°/0°. Here, 0° denotes that fibers are orientated along the loading direction. The overall dimensions of the GFC specimens were identical to the previously tested aluminum alloy specimens. The GFC specimens had 3 mm, 6 mm, and 9 mm diameter holes at their centers. Three specimens were prepared for the same hole size, and all the GFC specimens were tested under tensile loading until failure. 

### 6.2. Multiscale Failure Modeling

**Figure 21:**
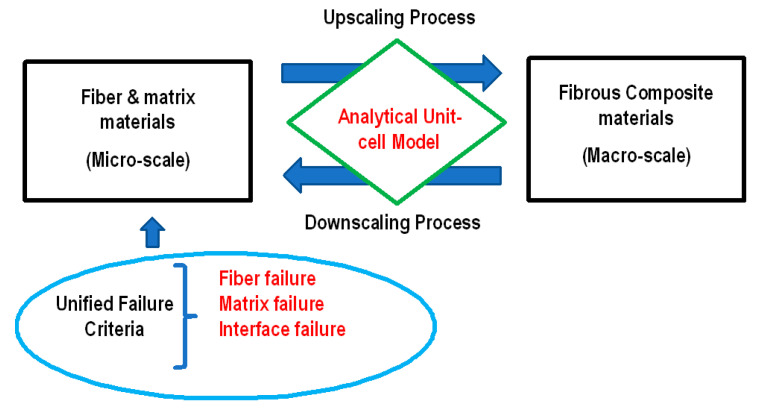
Multiscale approach.

Failure of laminated composite structures was modeled using a multiscale approach, which links the microscale and the macroscale of the composite materials and structures. The microscale indicates the fiber and matrix materials, while the macroscale is the smeared or homogenized composite material. The multiscale approach is sketched in Figure 21, and it consists of two processes bridging the microscale and macroscale. The first process is to transfer the information at the microscale to that at the macroscale. This process, called the upscaling or stiffness process, computes the effective composite material properties from the material properties of the fiber and matrix and their volume fractions. The second process occurs in the opposite direction and transfers information from the macroscale down to the microscale. This is called the downscaling process or the strength process. This second process determines the stresses and strains in the fiber and matrix materials from those at the composite material level. The main reason for this is to apply failure criteria at the fiber and matrix material level. The three different failure modes at the microscale are fiber failure, matrix failure, and fiber/matrix interface failure; for example, interlaminar delamination is described as matrix failure and/or fiber/matrix interface failure.

The overall analysis occurs in the following manner.

1. First, the composite material properties are computed from virgin fiber and matrix materials using the upscaling process.
2. The composite material properties are used for the analysis of the given composite structure with an applied loading. Because the structural analysis is complex, FEA is mostly used for the structural analysis, which provides the stresses and strains in the composite structure.
3. Then, the composite level stresses and strains are decomposed into the stresses and strains at the fiber and matrix materials using the downscaling process.
4. The unified failure criteria are applied to the stresses and strains of the fiber and matrix materials.
5. If there is a failure, then the corresponding material properties are degraded based on the specific failure, and the degraded material properties are used for the next upscaling process.
6. The analysis cycle repeats as failure progresses locally or the applied load increases.

Because both upscaling and downscaling processes are used iteratively, the computational cost of the multiscale analysis may be quite high. To overcome this, both upscaling and downscaling processes use analytical solutions without any additional numerical model. The derivations of the analytical solutions are based on a unit cell model as described in Refs. [40,41]. The unit cell consists of subcells. Some of the subcells represent the embedded fibers, and the remaining subcells represent the surrounding matrix material. Stresses and strains were assumed constant with every subcell for mathematical simplicity. Then, stress equilibrium and deformation compatibility are applied to the subcells to derive the equations necessary for the upscaling and downscaling processes. The details of the analytical derivations for the up and downscaling processes are omitted here. In summary, the upscaling process has the following analytical expression:

(5) $$E_{ijkl}^{c}=f\left(E_{ijkl}^{f},E_{ijkl}^{m},\nu^{f},\nu^{m}\right)$$

where *E_ijkl_* is the material property tensor; *ν* is the volume fraction; and superscripts ‘*c*’, ‘*f*’, and ‘*m*’ denote the homogenized composite, fiber, and matrix materials, respectively. This equation computes t homogenized composite material properties directly from the fiber and matrix material properties. 

The analytical expression used for the downscaling process is expressed as below:

(6) $$\varepsilon_{ij}^{f}=g_{1}\left(\varepsilon_{ij}^{c}\right)\;\mathrm{and}\;\varepsilon_{ij}^{m}=g_{2}\left(\varepsilon_{ij}^{c}\right)$$

in which *ε_ij_* is the strain tensor, and the same superscripts as before were used. Once the strains at the fiber and matrix materials are determined from Equation (6), the stresses at the fiber and matrix materials are computed as below:

(7) $$\sigma_{ij}^{f\;\mathrm{or}\;m}=E_{ijkl}^{f\;\mathrm{or}\;m}\varepsilon_{ij}^{f\;\mathrm{or}\;m}$$

where *σ_ij_* is the stress tensor. 

The failure criteria at the microscale level are given for the fiber breakage/buckling, matrix cracking, and fiber/matrix interface debonding. Fiber failure is the major catastrophic failure of fibrous composites because fibers are the major load-carrying elements. The effective stress for the fiber failure is expressed as

(8) $$\sigma_{e}^{f}=\sqrt{{\left(\sigma_{11}^{f}\right)}^{2}+{\left(\frac{E_{11}^{f}}{G_{12}^{f}}\right)}^{2}\left[{\left(\sigma_{12}^{f}\right)}^{2}+{\left(\sigma_{13}^{f}\right)}^{2}\right]}$$

This effective stress is applied to the failure criterion based on the stress and stress gradient as given in Equations (1) and (2). In Equation (8), ‘1’ is the fiber orientation, and ‘2’ is the transverse direction normal to the fiber orientation. In addition, *E* and *G* are elastic and shear moduli. All the components in Equation (8) are for the fiber material.

The matrix material used in this study is an isotropic brittle material, so as a result, the maximum normal stress in the matrix material is used for the effective stress. Finally, the effective stress to check the fiber/matrix interface failure is given below:

(9) $$\sigma_{e}^{\mathrm{int}}=\sqrt{{\left(\frac{\sigma_{12}^{m}+\sqrt{v^{f}}\left(\sigma_{22}^{m}-\sigma_{11}^{m}\right)}{\tau_{\mathrm{fail}}^{\mathrm{int}}}\right)}^{2}+{\langle \frac{\sigma_{22}^{m}}{\sigma_{\mathrm{fail}}^{\mathrm{int}}}\rangle }^{2}}$$

where $\tau_{\mathrm{fail}}^{\mathrm{int}}$ and $\sigma_{\mathrm{fail}}^{\mathrm{int}}$ are the tangential and normal failure strength of the interface. In addition, <…> in Equation (9) is the Macaulay function. Hence, this function is used to indicate that only the tensile but not compressive normal stress at the fiber/matrix interface contributes to the interface failure.

### 6.3. Results

The GFC specimens behaved like quasi-brittle material. In order to view the failure surfaces closely, GFC specimens were sputter-coated with approximately 15 nm of Pt/Pd. Because GFC is non-conductive, it must be coated with a conductive metal to achieve high-quality images using scanning electron microscopy. Additionally, sputter coating the GFC samples prevents the GFC from absorbing the energy, which could result in deforming the samples.

**Figure 22:**
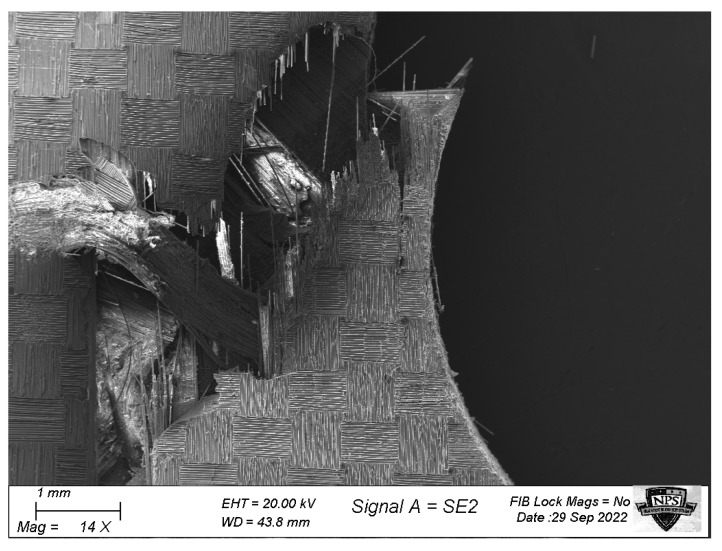
Left side edge of fracture of the GFC specimen with a hole.

**Figure 23:**
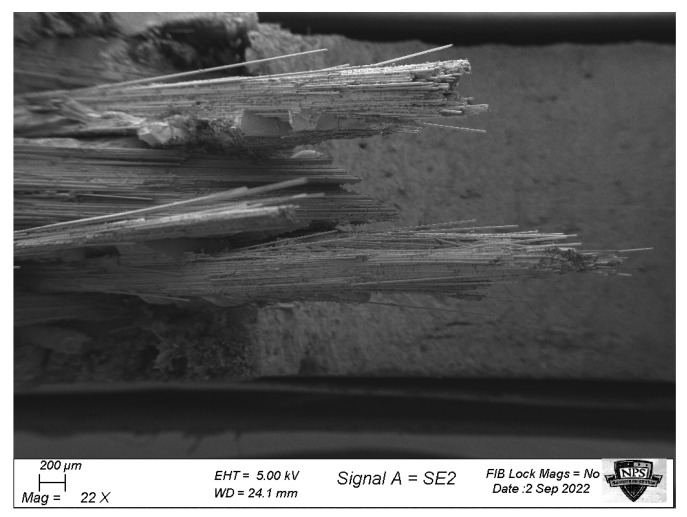
Fiber pull-out failure of ±45° fibers of the GFC specimen with a hole.

Figure 22 shows that the GFC samples, regardless of hole size, had fiber fractures in the 0° layers at the edge of the hole with minimal cross-section. The fiber fracture initiated in the perpendicular direction to the applied loading, and then it propagated at approximately 45°. On the other hand, the ±45° layers showed no fiber fracture but indicated that fiber pull-out had occurred, as shown in Figure 23. The failure of the ±45° layers occurred after the failure of the 0° layer. Thus, the applied failure stresses of the GFC specimens were obtained at the onset of the initial fiber fracture of the 0° layers. 

The multiscale analysis, as described in the previous section, was conducted for the GFC specimens with holes. As the applied load increased incrementally, failure criteria at the fiber and matrix material levels were checked, respectively, using the effective stress and the stress gradient condition. Then, when the 0° layers initiated the fiber fracture at the edge of each hole, it was the onset of the main failure. The applied failure stress was then determined at that applied load. 

**Figure 24:**
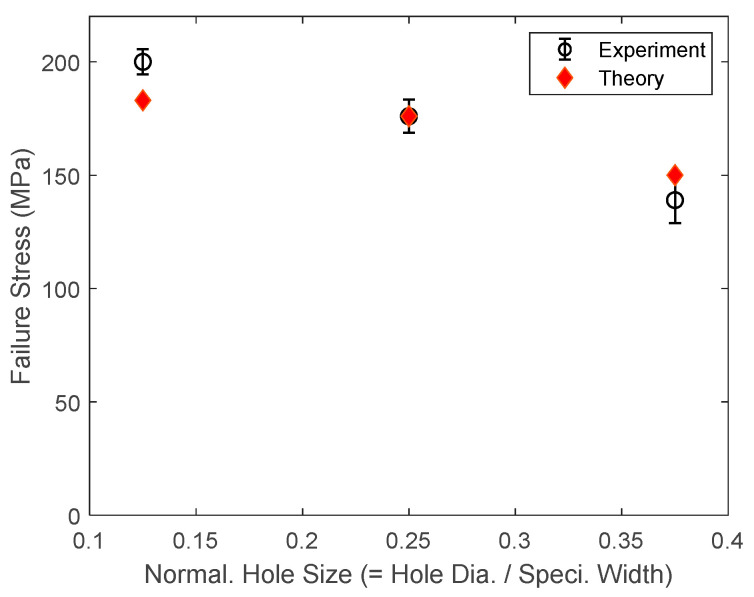
Comparison of experimental and theoretical failure stresses of GRP specimens with center holes.

Figure 24 shows the comparison between the experimental and predicted failure stresses, which were computed from the applied load divided by the specimen cross-section at the grip locations, i.e., the section without holes. Because the fiber failure value Υ for the stress-gradient condition was obtained from the 6 mm hole specimens, the theoretical prediction is exactly on top of the mean experimental failure stress. Using the same failure value, failure stresses were predicted for the specimens with a 3 mm or 9 mm hole. As shown in Figure 24, the theoretically predicted failure stresses agreed well with the experimental stresses, which also suggests that the unified failure criterion in association with the multiscale approach is useful for predicting the failure of the quasi-brittle laminated composites.

## 7. Summary and Conclusions

A new unified failure criterion was proposed to predict the failure loads of structural members. The criterion uses both stress and stress gradient to determine failure. For failure to occur, both stress and stress gradient conditions must be satisfied simultaneously. Various scenarios were examined to validate the new failure criterion. The cases included brittle or quasi-brittle materials and a ductile aluminum alloy. The former materials were isotropic hardened cement pastes, orthotropic PLA, and laminated glass fiber composites. Different geometric features were also tested, including a crack, a long slit, and a circular hole of different sizes. The new failure criterion predicted the failure loads satisfactorily for all the cases examined in this study as compared to their corresponding experimental results. The failure criterion showed that the failure load was ultimately determined by either the stress condition or stress-gradient condition depending on the material or geometric conditions of the tested specimens, even though both conditions were satisfied simultaneously for all the specimens.

## Figures and Tables

**Table 1:** Nominal failure stress of aluminum specimens with different sizes of holes.

Hole Size (mm)	0	1	2	3	6	9	12	15	18
Average Failure Stress (MPa)	228	237	236	228	236	219	227	228	230
Standard Deviation (MPa)	5.5	1.6	1.3	3.2	3.7	4.8	0.94	1.8	0.96

**Table 2:** Printing conditions for PLA specimens.

Print temperature	185 °C
Bed temperature	55 °C
Print speed	45 mm/s
Line thickness	0.2 mm
Line width	0.35 mm

## Data Availability

All the data were provided in this paper in the form of figures and tables.
